# The β-catenin/TCF-4 pathway regulates the expression of OPN in human osteoarthritic chondrocytes

**DOI:** 10.1186/s13018-020-01881-6

**Published:** 2020-08-20

**Authors:** Jian Tian, Shu-Guang Gao, Yu-Sheng Li, Chao Cheng, Zhen-Han Deng, Wei Luo, Fang-Jie Zhang

**Affiliations:** 1 Department of Orthopaedics, Xiangya Hospital, Central South University, No.87 Xiangya Road, Changsha, 410008 Hunan China; 2 Department of Orthopaedics, Yiyang Central Hospital, Clinical Medical Technology Demonstration Base for Minimally Invasive and Digital Orthopaedics in Hunan Province, No.118 North KangFu Road, Yiyang, 413000 Hunan China; 3 Department of Sports Medicine, The First Hospital Affiliated to Shenzhen University, Shenzhen Second People’s Hospital, Shenzhen, 518035 Guangdong China; 4 Department of Emergency Medicine, Xiangya Hospital, Central South University, No.87 Xiangya Road, Changsha, 410008 Hunan China; 5 National Clinical Research Center for Geriatric Disorders, Xiangya Hospital, Central South University, No.87 Xiangya Road, Changsha, 410008 Hunan China

**Keywords:** β-Catenin, Chondrocyte, Dickkopf 1, Osteopontin, TCF 4

## Abstract

**Background:**

Cartilage destruction is the main characteristic of osteoarthritis (OA), and osteopontin (OPN) is elevated in OA articular cartilage; however, the reason for the increased OPN level is not determined. In addition, Wnt/β-catenin signaling participates in the progression of OA. The aim of the present study was to evaluate whether canonical Wnt signaling could regulate the expression of OPN in human chondrocytes in vitro.

**Methods:**

Human chondrocytes were cultured in vitro, and we first assayed the mRNA levels of OPN and β-catenin in chondrocytes. Next, we performed transient transfection of TCF 4 shRNA into chondrocytes to inhibit TCF 4 expression and explore changes in the OPN level. Then, the Wnt/β-catenin signaling inhibitor Dickkopf-1 (Dkk-1) was incubated with chondrocytes, and we assayed the changes in β-catenin and OPN.

**Results:**

Our results showed that the expression of both β-catenin and OPN was increased in OA chondrocytes, but there were no correlations between β-catenin and OPN expression. TCF4 shRNA downregulated the expression of TCF 4 and OPN in chondrocytes, while after treatment with rDKK-1 at a concentration of 400 ng/ml for 24 h, the mRNA and protein expression of both β-catenin and OPN was significantly decreased in chondrocytes.

**Conclusions:**

Elevated OPN expression might be regulated by the β-catenin/TCF-4 pathway, and the Wnt/β-catenin inhibitor DKK1 could inhibit the expression of β-catenin and OPN in OA chondrocytes.

## Introduction

Osteoarthritis (OA) is the leading reason for disability and mainly affects the elderly [1]. Radiological evidence shows that OA occurs in most people over 65 years of age and approximately 80% of people over 75 years of age [2]. OA, which is characterized by articular cartilage destruction and changes in other joint components, including bone, meniscus, synovium, ligaments, and muscles, can affect all structures of the entire joint [1]. The pathogenesis of OA involves multiple factors, including mechanical, genetic, and aging-related factors, which ultimately lead to synovitis, apoptosis, and cartilage destruction [3–5].

Osteopontin (OPN) is a 44–75 kDa multifunctional phosphoprotein secreted by numerous cell types, including osteoclasts, chondrocytes, synoviocytes, macrophages, and epithelial cells [6–8]. A previous study indicated that the levels of OPN in plasma, synovial fluid, and articular cartilage are associated with progressive joint damage and are likely to be a useful biomarker for determining disease severity and progression in knee OA [9, 10]. Other studies confirmed that OPN could promote the production of MMP13 and activation of the NF-kappaB pathway by increasing the abundance of p65 and phosphorylated p65 and the translocation of the p65 protein from the cytoplasm to the nucleus in chondrocytes [11]. In OA progression, OPN plays an important role as an intrinsic regulator. Previous studies have attempted to use this protein as a diagnostic marker of OA and to use OPN as a target for drug development against OA [12]. However, until recently, the main reason for the increased expression of OPN in OA was undetermined.

The Wnt/β-catenin signaling pathway can directly affect the bone and bone and synovial tissues to play a role in bone and joint pathology. Wnt/β-catenin proteins affect cell stability by regulating cell proliferation and determining cell fate and the differentiation state [13, 14]. The development of joints, including the cone, bone, and joint cavities, is highly dependent on the direction of Wnt signaling. Historically, Wnt signaling pathways have been divided into β-catenin-dependent classical Wnt signaling pathways and various β-catenin-independent noncanonical pathways [15]. Drugs that increase β-catenin activity may have the potential to treat OA by reducing articular cartilage degeneration [14, 16]. The T cell factor (TCF) transcription factor is a downstream effector restored by Wnt/β-catenin signaling and is related to the occurrence and development of OA. Overexpression of TCF4 in OA cartilage can induce MMP-1, MMP-3, and MMP-13 expression and general MMP activity and induce human chondrocyte apoptosis [17]. Dickkopf-1 (Dkk-1), an inhibitor of the Wnt/β-catenin signaling pathway, is also involved in the regulation of capillary interactions and bone formation [18]. The expression of Dkk-1 in plasma and synovial fluid is inversely related to the severity of knee OA joint injuries [19]. Increased Dkk-1 can inhibit classical Wnt signaling dysregulation, thereby reducing the induction of MMP 3 and IL-6 and inhibiting OA helix destruction in experimental OA models [20, 21].

Previous studies have confirmed that OPN is transformed by a variety of Wnt signaling factors, including TCF4 and β-catenin [22, 23]. High expression of TCF4 results in OPN promoter activity and protein expression in rat mammary carcinoma cells [24], and TCF4 can target repressors or activators of metabolic progression by regulating OPN expression via Wnt restoration [25]. However, the relationship between Wnt/β-catenin signaling and OPN in human chondrocytes is inconclusive. Therefore, in this study, we evaluated whether classical Wnt signaling can regulate OPN expression in human chondrocytes in vitro.

## Materials and methods

### Human chondrocyte culture

**Fig. 1:**
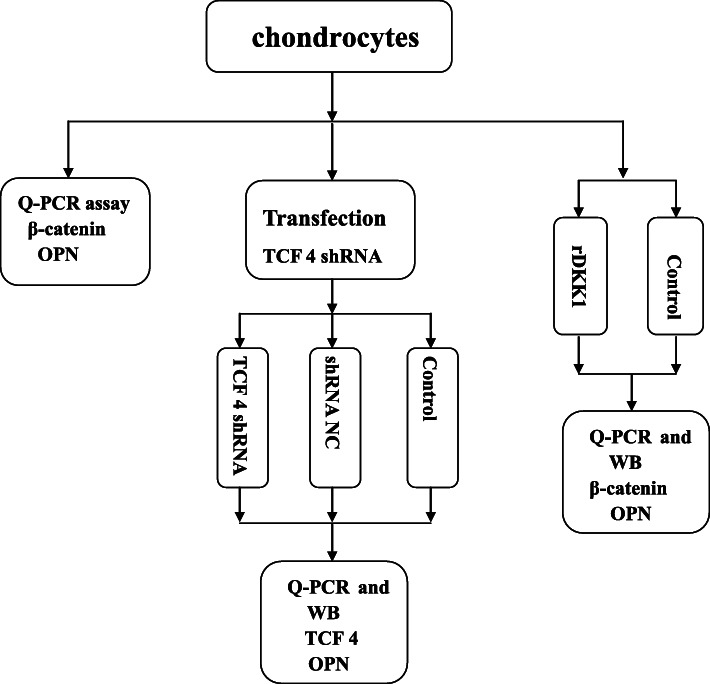
The experimental flowchart

The concise experimental flowchart is shown in Fig. 1. The study protocol was approved by the Ethics Committee of Xiangya Hospital, Central South University. Each participant or the legally authorized representative of the participant was aware of and agreed to the study. Only cartilage samples from unique donors were employed for each culture. Thus, for each experiment, cells were obtained from the same subject. Normal human articular cartilage was obtained from five subjects (aged 14–50 years) who underwent knee amputation because of severe trauma. OA human articular cartilage was obtained from 11 patients (aged 59–75 years) with knee OA who were undergoing total knee replacement surgery. After washing twice with phosphate-buffered saline (PBS), the cartilage tissue was ground with a scalpel blade into 1–5 mm^3^ sections. The cartilage tissue was subsequently digested with 5–8 ml of 0.2% collagenase II (Sigma-Aldrich, St. Louis, MO, USA) for 12–16 h at 37 °C in a constant temperature shaker. Digestion was terminated with 8–10 ml of Dulbecco’s modified Eagle’s medium/F12 (DMEM/F12; HyClone, Logan, UT, USA). The released cell pellets at the bottom of the centrifuge tube were aspirated and transferred to a culture flask following centrifugation at 1000 rpm for 5 min. Cell pellets were resuspended in 5 ml of DMEM/F12 containing 10% fetal bovine serum (FBS; Gibco, Grand Island, NY, USA) and 1% penicillin/streptomycin solution (Gibco) and incubated for 24 h at 37 °C with 5% CO_2_ in a plastic culture flask. The nonadherent cells were subsequently washed out. The growth medium was changed every 3 days prior to trypsinization, and cells were then passed to a new 6-well plate at a density of 5 × 105 cells per well. Cells at passages one through two were used for experiments.

### Total RNA isolation, quantification, and reverse transcription; real-time quantitative PCR assays

Total cellular RNA was extracted from cultured normal and osteoarthritic chondrocytes using TRIzol reagent (Invitrogen, Life Technologies, Paisley, UK). RNA was further purified using an RNeasy mini kit (Qiagen, Hilden, Germany) according to the manufacturer’s instructions. Preservation of 28S and 18S ribosomal RNA (rRNA) species was used to assess RNA integrity. All samples included in the study had prominent 28S and 18S rRNA components. The total RNA yield was quantified by spectrophotometry. One microgram of total RNA was converted to cDNA using a Revert Aid™ First Strand cDNA Synthesis Kit (Fermentas, Thermo Fisher Scientific, Waltham, MA, USA). First, all components including template RNA (1 μg) and oligo (dT)18 primer (1 μl) were mixed, and nuclease-free water was added to a total volume of 12 μl. The mixture was mixed gently, centrifuged briefly, and incubated at 65 °C for 5 min. The mixture was chilled on ice, spun down, and placed back in a vial on ice. The following components were added, to a volume of 20 μl: 5X reaction buffer (4 μl), RiboLock™ RNase Inhibitor (20 u/μl) (1 μl), 10 mM dNTP Mix (2 μl) and Revert Aid™ M-MuLV Reverse Transcriptase (200 u/μl) (2 μl). This reaction was then incubated for 60 min at 42 °C and terminated by heating at 70 °C for 5 min. The resulting cDNA products were stored in aliquots at − 80 °C until needed.

The primers were synthesized by Shanghai Sangon Bioengineering Corporation. All primers used are shown in Table 1. For all real-time quantitative PCR reactions, Maxima® SYBR Green/ROX qPCR Master Mix (2×) (Fermentas, Thermo Fisher Scientific, Waltham, MA, USA) was used (12.5 μl of Maxima® SYBR Green/ROX qPCR Master Mix (2×), 2.5 μl of forward primer (0.3 μM), 2.5 μl of reverse primer (0.3 μM), and 2 μl of template DNA and 5.5 μl of nuclease-free water were added to a volume of 25 μl). The ABI 7900 Sequence Detection System (Applied Biosystems, Foster City, CA, USA) was used for all real-time Q-PCRs. The PCR thermal cycling protocol applied consisted of 1 step for 2 min at 50 °C for UDG pretreatment and 1 step for 10 min at 95 °C for initial denaturation followed by 40 cycles consisting of a denaturation step for 15 s at 95 °C, an annealing step for 30 s at 30 °C, and an extension step for 30 s at 72 °C. A melting curve analysis was performed after the final amplification period with a temperature gradient of 95 °C for 15 s, 60 °C for 15 s, and 95 °C for 15 s.

**Table 1:** Oligonucleotide primers used in real-time PCR assay

Gene	Forward primer sequence	Reverse primer sequence
β-catenin	5′-GAGGAGATGTACATTCAGCAG-3′	5′-GTCTCCGACCTGGAAAAC-3′
TCF-4	5′-CTTTCCCTAGCTCCTTCTTC-3′	5′-CTACGATGGAAAGTGGACAT-3′
OPN	5′-GTGGGAAGGACAGTTATGAA-3′	5′-CTGACTTTGGAAAGTTCCTG-3′
GAPDH	5′-CAATGACCCCTTCATTGACC-3′	5′-GACAAGCTTCCCGTTCTCAG-3′

Real-time Q-PCR test results: The relative expression level of mRNAs was calculated as the ratio of each mRNA expression level to that of GAPDH mRNA as the reference housekeeping gene. The relative expression levels of genes of interest were calculated and expressed as 2^−△△CT^ values. All quantities were expressed as *n*-fold relative to a calibrator.

### Transient transfection with TCF-4 shRNA in chondrocytes

siRNAs specific to TCF-4 based on the coding sequence of human TCF-4 were designed and synthesized by GenePharma Corporation (China, Shanghai). The three shRNA sequences and scrambled shRNA sequences constructed are shown in Table 2. Normal and osteoarthritic chondrocytes were seeded in six-well plates at a density of 5 × 105 cells/well in a medium without antibiotics. After overnight incubation, when cells reached 70% confluence, they were transfected with specific shRNAs against TCF-4 with Lipofectamine TM 2000 reagent (Invitrogen, San Diego, CA, USA) according to the manufacturer’s instructions. No cell toxicity from the transfection agent was detected. The transfection efficiency was evaluated with fluorescein by fluorescence microscopy 24 h after transfection. In each experiment, the results from three of the six-well plates were averaged and considered *n* = 1. No significant variance was observed among the individual wells in each averaged group. After 24 h, total RNA and protein were isolated, and the expression levels of TCF-4 and OPN were detected by real-time Q-PCR and Western blot analyses.

**Table 2:** TCF-4 siRNA sequences used in transient transfection

	Sense	Antisense
siRNA-1105	5′-GCCATGGAGGTACAGACAAAGTTCA-3′	5′-GAGACTTTGTCTGTTACCTCCATGGCTT-3′
siRNA-1791	5′-GGATGATGCTATTCATGTTCTTTCA-3′	5′-GAGAAGAACATGAATAGCACTACATCCTT-3′
siRNA-1859	5′-GGGACATGCATGGAATCATTGTTCA-3′	5′-GACACAATGATTCCATGCATGTCCCTT-3′
NC-siRNA	5′-GTTCTCCGAACGTGTCACGTCAAG-3′	5′-GATTACGACACGTTCGGAGAATT-3′

### Cell treatment with recombinant DKK-1

Normal and OA chondrocytes were seeded on six-well plates at 1 × 106 cells/well, and 3 days after seeding, the cells were treated with recombinant DKK-1 (100, 200, 300, 400, and 500 ng/ml) for 12, 24, 36, and 48 h; each experiment was conducted with triplicate wells.

Cell viability was determined by a colorimetric 3-(4,5-dimethylthiazol-2-yl)-2,5-diphenyltetrazolium bromide (MTT) assay. The culture medium was removed, and 20 μl of MTT solution (5 mg/ml in PBS) was added to each well and incubated at 37 °C with 5% CO_2_ for 4 h. The supernatant was then carefully aspirated, and the formazan reaction products were dissolved in 150 μl dimethylsulfoxide (DMSO) (Sigma, St Louis, MO, USA) solution and shaken for 15 min. The spectrophotometric absorbance at 570 nm was measured in an enzyme-linked immunosorbent assay (ELISA) plate reader (Multiskan MK3-Thermo Labsystems; Thermo Fisher Scientific, Waltham, MA, USA) or an ELISA reader (Bio-Rad, CA, USA).

### Western blot analysis

Chondrocytes were lysed by using RIPA buffer and a cocktail of protease and phosphatase inhibitors. The protein concentration was quantified by using a BCA Protein Assay Kit (Thermo Scientific Company, Prod#23225, Rockford, USA) with bovine serum albumin as the standard. Cell lysates from normal and OA chondrocytes were electrophoresed and separated on a 4 to 20% Tris-HCl gel (Bio-Rad, Hercules, CA, USA), and the separated proteins (25 μg) were electrotransferred to polyvinylidene fluoride membranes (Millipore, Bedford, MA, USA). Nonspecific proteins on the membranes were blocked with 5% skim milk powder in PBS. The membrane was probed with an anti-osteopontin antibody (ab8448, 1:1,000 dilution, Abcam, Cambridge, MA, USA) and an anti-TCF-4 antibody (sc-57040; Santa Cruz Biotechnology, Santa Cruz, CA, USA) overnight at 4 °C. A mouse monoclonal anti-actin antibody (Sigma) was used as the loading control. The membranes were then incubated with the appropriate horseradish peroxidase (HRP)-conjugated secondary antibodies (1:10,000 dilution). Immunoreactive proteins were visualized with western blotting luminol reagent (Santa Cruz Biotechnology, Santa Cruz, CA, USA). The polyvinylidene fluoride membranes were then exposed to photographic film, which was scanned, and the intensities of the protein bands were determined with computerized densitometry. The results were normalized by using an anti-GAPDH polyclonal antibody (Sigma-Aldrich, St. Louis, MO, USA).

### DAPI staining

β-Catenin nuclear accumulation was observed by nucleic acid staining with DAPI. Chondrocytes were treated with or without rDKK 1, and the treated chondrocytes were collected and fixed in 4% paraformaldehyde for 5 min. The fixation solution was discarded, and the cells were rinsed 3 times in 1× PBS. The buffer was discarded, and the cells were incubated in 10 μM DAPI solution in the dark at 37 °C for 15 min. The DAPI solution was removed, and the stained cells were rinsed 3 times in 1× PBS and supplemented with fresh 1× PBS buffer. The stained cells were examined for cellular morphology and nuclear profiles under a Leica laser confocal microscope.

### Statistical analysis

All statistical calculations were performed using GraphPad Prism 6.0 (GraphPad Software, Inc., La Jolla, CA, USA). Data are expressed as the means ± standard errors of the mean. Student’s *t* test was used to analyze statistical differences between two groups, and one-way ANOVA was performed to determine the statistical differences among groups. A *P* value of less than 0.05 was considered statistically significant.

## Results

### Overexpression of β-catenin and OPN mRNA in OA chondrocytes

**Fig. 2:**
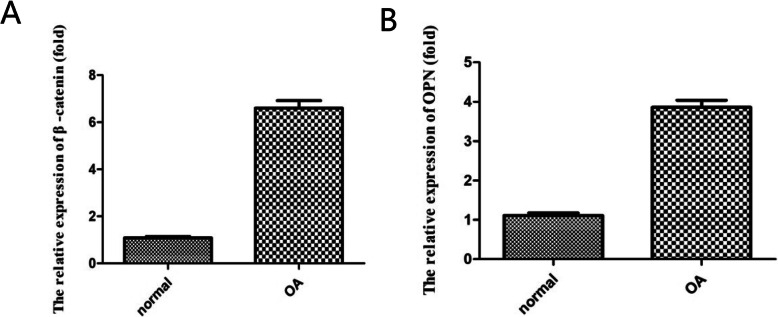
**a** β-catenin mRNA expression was significantly higher in the OA group than in the normal group. **b** OPN mRNA expression was significantly higher in the OA group than in the normal group

In the present study, we detected the mRNA levels of β-catenin and OPN between chondrocytes isolated from parts of the knee cartilage of OA and non-OA patients. The relative β-catenin mRNA expression in the OA group (6.60 ± 0.31-fold) was significantly higher than that in the normal group (1.08 ± 0.05-fold) (*P* < 0.01) (Fig. 2a). We also found significant upregulation of OPN mRNA in OA chondrocytes (3.85 ± 0.18-fold) compared to normal chondrocytes (1.11 ± 0.06-fold) (*P* < 0.001) (Fig. 2b). However, the expression of β-catenin mRNA did not correlate with the OPN mRNA level in OA chondrocytes (Pearson *r* = 0.2447, *P* = 0.4683, *P* > 0.05).

### Transient transfection of TCF-4 shRNA into chondrocytes

**Fig. 3:**
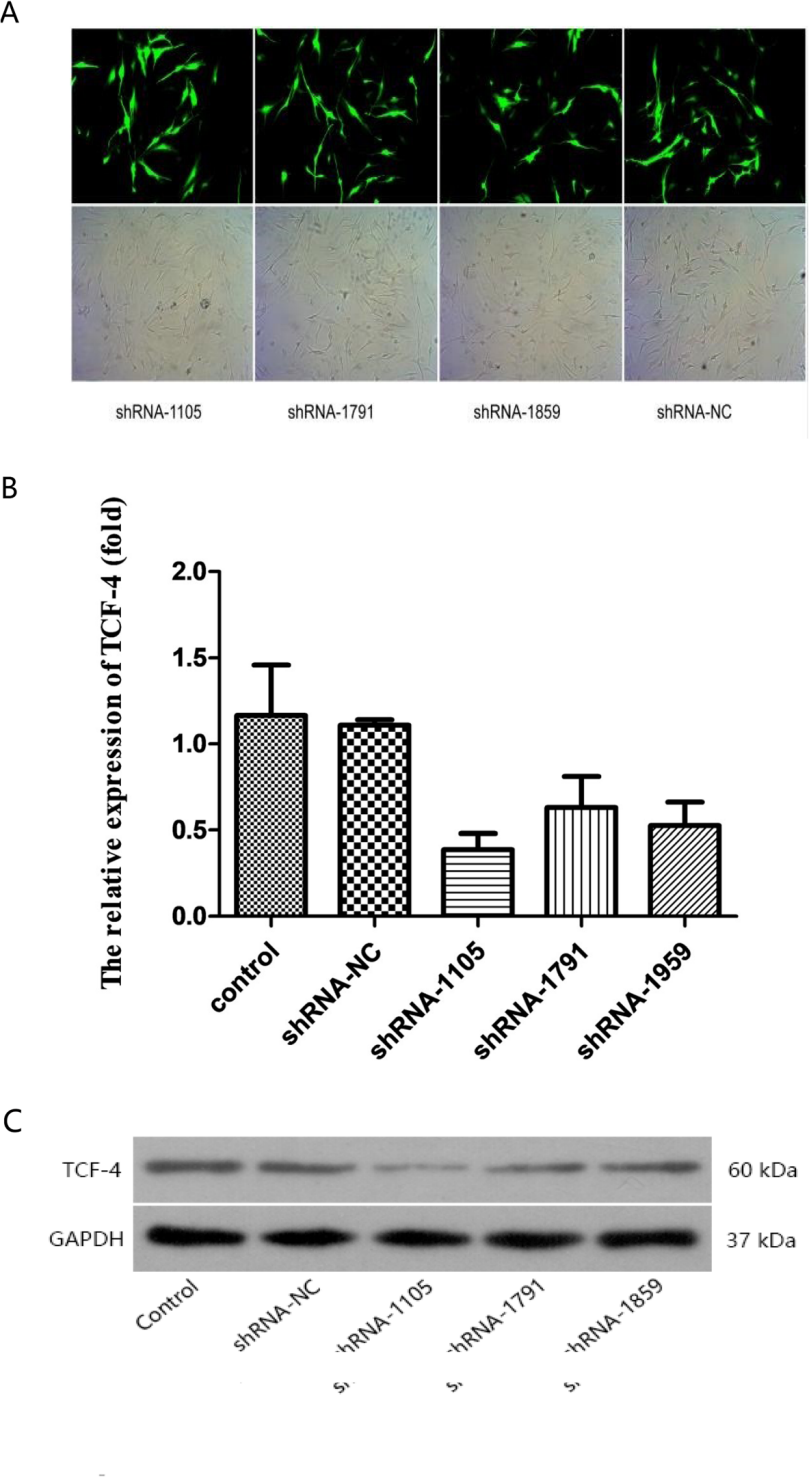
**a** The transfection efficiency of each shRNA sequence after transfection for 24 h. **b**, **c** Compared with other shRNA sequences, shRNA 1105 was the most effective at decreasing TCF4 expression

To determine which siRNA sequences could maximally decrease TCF-4 expression, three paired sequences (shRNA1105, shRNA1791, and shRNA1959) and one negative control sequence (shRNA NC) were transiently transfected into OA chondrocytes. The transfection efficiency was evaluated with fluorescein by fluorescence microscopy 24 h after transfection. Compared to the same optical field, the fluorescence index value for the transfected cells was nearly 50% (Fig. 3a), and the expression of TCF-4 mRNA and protein was detected by real-time Q PCR and western blotting, respectively. The results showed that shRNA-1105 decreased TCF-4 mRNA and protein expression more efficiently than the other two sequences (*P* < 0.05) (Fig. 3b, c); therefore, TCF4 shRNA-1105 was used in the following experiment.

### TCF4 shRNA-1105 downregulated the expression of TCF4 and OPN

**Fig. 4:**
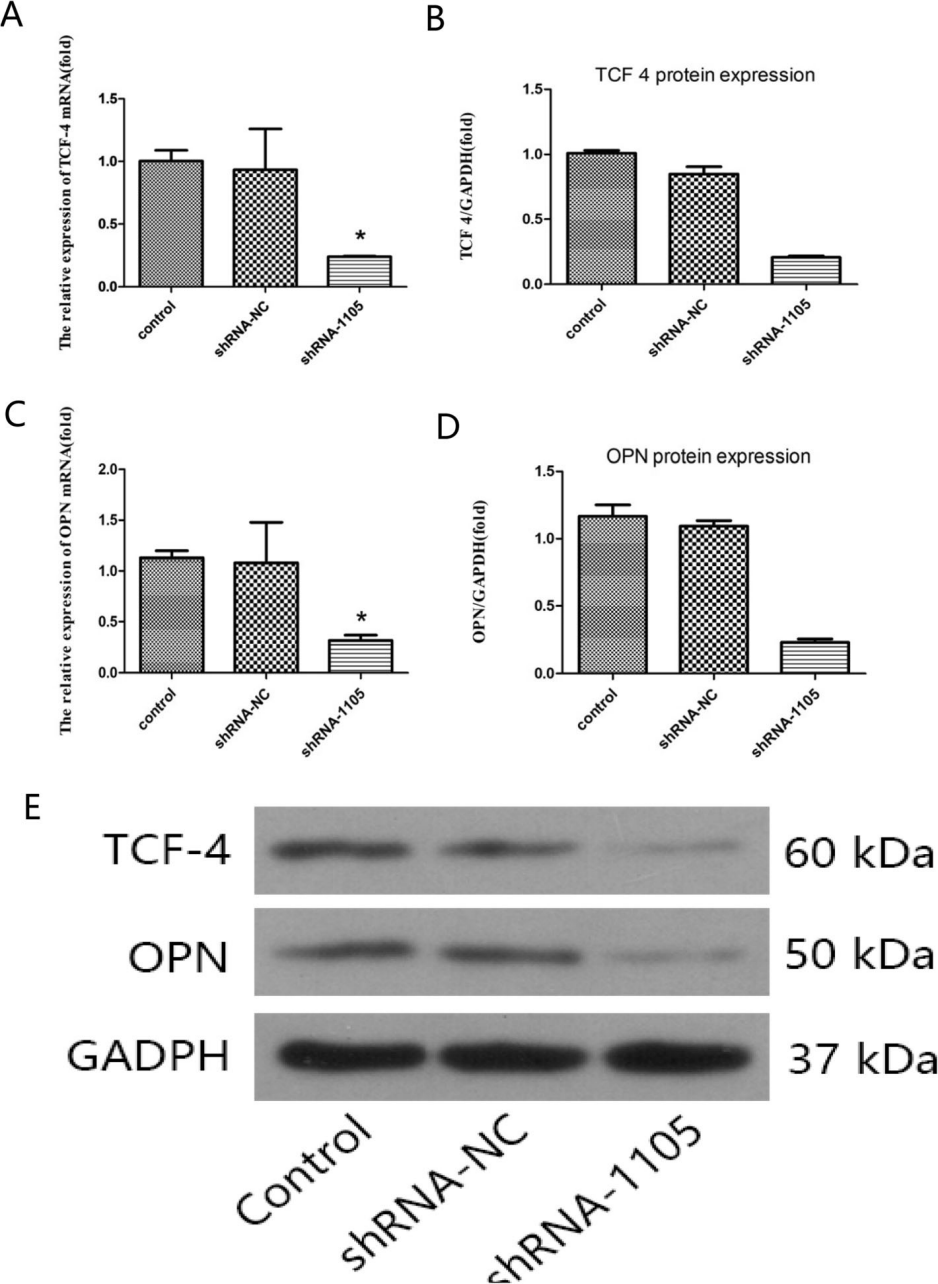
**a**, **b**, and **e** The decrease in TCF 4 expression after shRNA 1105 treatment. **c**–**e** The decrease in OPN expression after shRNA 1105 treatment

In this experiment, we treated chondrocytes in three groups: the control group, shRNA-1105 group, and shRNA NC group. All chondrocytes were treated for 24 h. Our results showed that the expression of TCF4 mRNA (0.21 ± 0.01-fold) and protein (0.20 ± 0.01-fold) was decreased in the shRNA-1105 group compared to the control group (*P* < 0.01) and shRNA NC group (*P* < 0.05) and that OPN mRNA (0.23 ± 0.01-fold) and protein (0.22 ± 0.02-fold) expression were decreased in the shRNA-1105 group compared to the control group (*P* < 0.01) and shRNA NC group (*P* < 0.05) (Fig. 4a–e).

### Effects of rDKK-1 treatment on chondrocyte viability

**Fig. 5:**
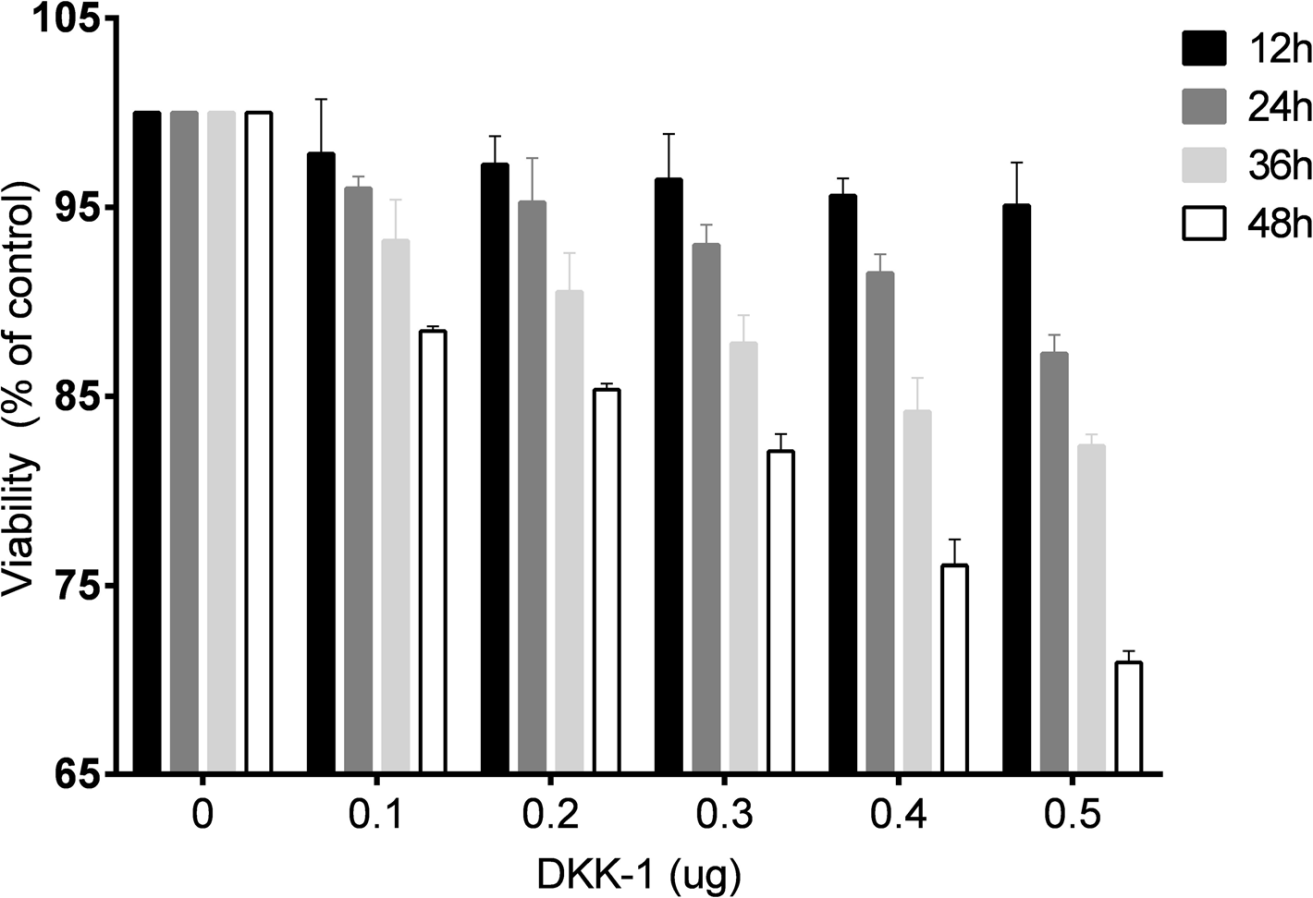
The effects of rDKK 1 on chondrocyte viability

In MTT assays, chondrocytes were treated with rDKK-1 at concentrations of 100, 200, 300, 400, and 500 ng/ml and for durations of 12, 24, 36, and 48 h to determine the adverse effects of rDKK-1 on cell viability. Our results showed that the relative cell viability was greater than 90% at 400 ng/ml for 24 h, as shown in Fig. 5. Therefore, to understand the impact of rDKK-1 on β-catenin and OPN, in subsequent experiments, cells were treated with DKK-1 at a concentration of 400 ng/ml for 24 h, and the levels of β-catenin were assayed by western blotting and Q-PCR.

### Effects of rDKK-1 on β-catenin and OPN expression in chondrocytes

**Fig. 6:**
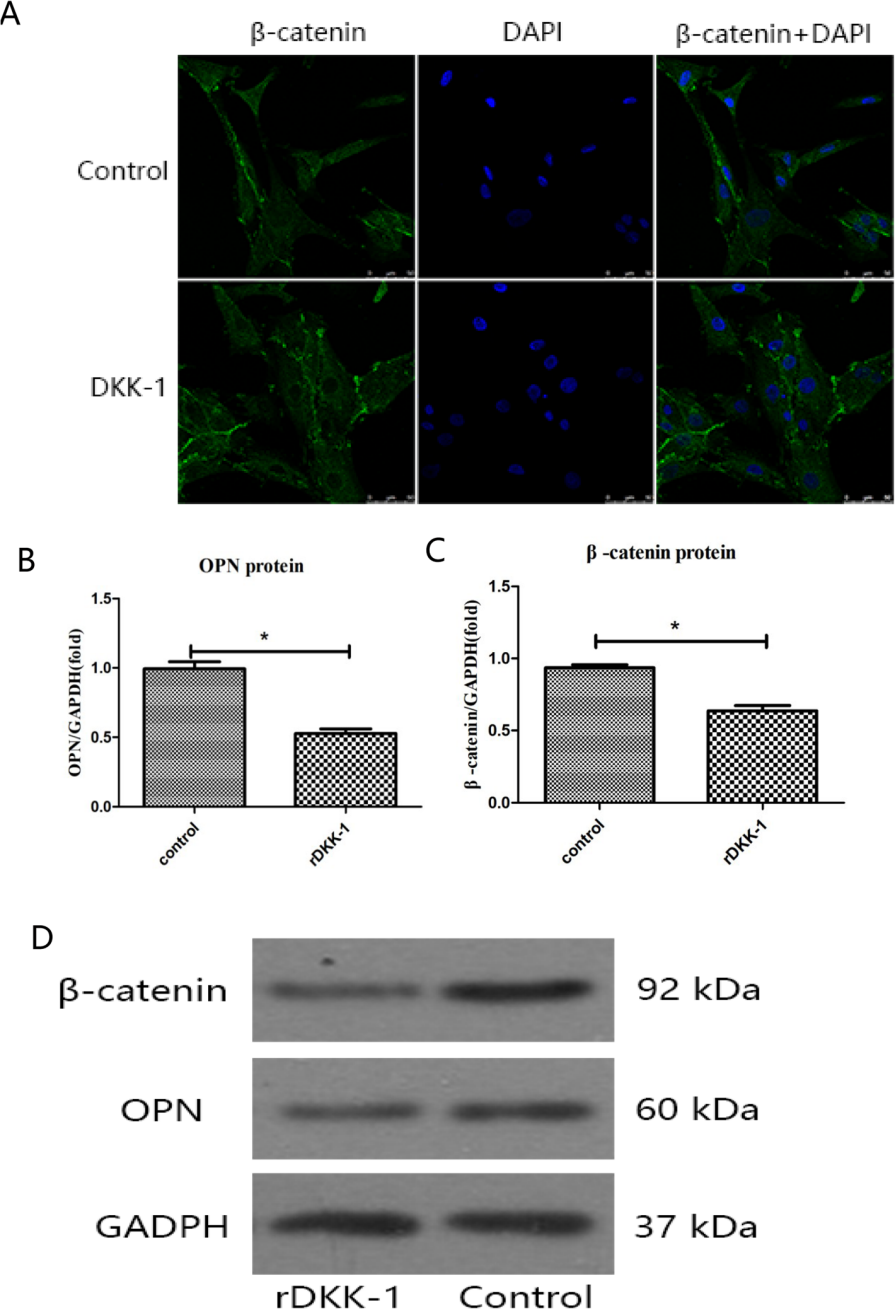
**a** β-Catenin nuclear accumulation was decreased by rDKK-1 treatment. **b**, **d** The decrease in OPN expression after rDKK-1 treatment. **c**, **d** The decrease in β-catenin expression after rDKK-1 treatment

It is well known that DKK 1 is an inhibitor of the Wnt/β-catenin signaling pathway, an our DAPI staining results indicated that β-catenin nuclear accumulation was decreased in the presence of rDKK-1, as shown in Fig. 6a. The mRNA and protein expression levels of both β-catenin and OPN were significantly decreased after chondrocytes were treated with rDKK-1 at a concentration of 400 ng/ml for 24 h, as shown in Fig. 6b–d (all *P* < 0.05).

## Discussion

Cartilage degeneration is the main feature of OA, and OPN is a secreted multifunctional phosphoprotein involved in the molecular pathogenesis of OA by contributing to progressive degeneration of articular cartilage [11]. In the present study, our results confirmed that the expression of OPN mRNA in human OA cartilage chondrocytes was enhanced compared with that in normal cartilage chondrocytes [7, 26]. Our previous study confirmed that OPN was elevated in OA cartilage and synovial fluid and played important roles in OA [9, 12]. In the past decade, many studies have focused on the role of OPN in OA progression, but the mechanisms by which OPN expression is regulated in OA are still incompletely understood, and few studies have focused on the reasons for the overexpression of OPN.

Canonical Wnt signaling is also called Wnt/β-catenin signaling; this pathway is a conserved signaling pathway implicated in the pathogenesis of OA [13, 20]. In the absence of Wnt, a destruction complex mediates the phosphorylation of β-catenin by glycogen synthase kinase-3β, which induces degradation of cytosolic β-catenin through the proteasome. The binding of Wnt to its receptors results in disruption of the destruction complex and accumulation of cytoplasmic β-catenin [27, 28]. Upon nuclear translocation, β-catenin functions as a cofactor for TCF/LEF transcription factors to switch on Wnt target gene transcription [29]. Our results indicated that the mRNA of β-catenin was increased in OA chondrocytes compared to normal chondrocytes, as shown in Fig. 2a, which proved that Wnt/β-catenin signaling was activated in OA chondrocytes; however, our results indicated that the expression of β-catenin did not correlate with the OPN level, although the levels of both β-catenin and OPN were increased in OA chondrocytes.

Mammals have four members of the TCF/LEF family: TCF1, TCF3, TCF4, and LEF1 [30]. Via alternative splicing and promoter use, each member is produced as an isoform. The N-terminal β-catenin binding domain is highly conserved and is responsible for β-catenin binding. Certain dependent regulatory domains and C-terminal tail sequences differ between all the four members, resulting in different binding characteristics. The regulatory region of the human OPN promoter has been shown to contain a TCF-4 binding site [31]. The expression of TCF 4 mRNA was found to be higher in OA chondrocytes than in normal chondrocytes, in accordance with a previous study [17]. In the present study, we first constructed a TCF-4 shRNA transient transfection system in OA chondrocytes; subsequently, the most efficient TCF4 shRNA-1105 was used for interference in OA chondrocytes to determine the effect of TCF 4 on the expression of OPN. Our results indicated that TCF4 shRNA-1105 could downregulate the expression of OPN, consistent with other studies reporting that TCF 4 can regulate OPN expression in a Wnt-dependent manner to act as a repressor or activator in breast cancer progression [25] and that the β-catenin/TCF4 transcriptional complex regulates OPN expression [23].

**Fig. 7:**
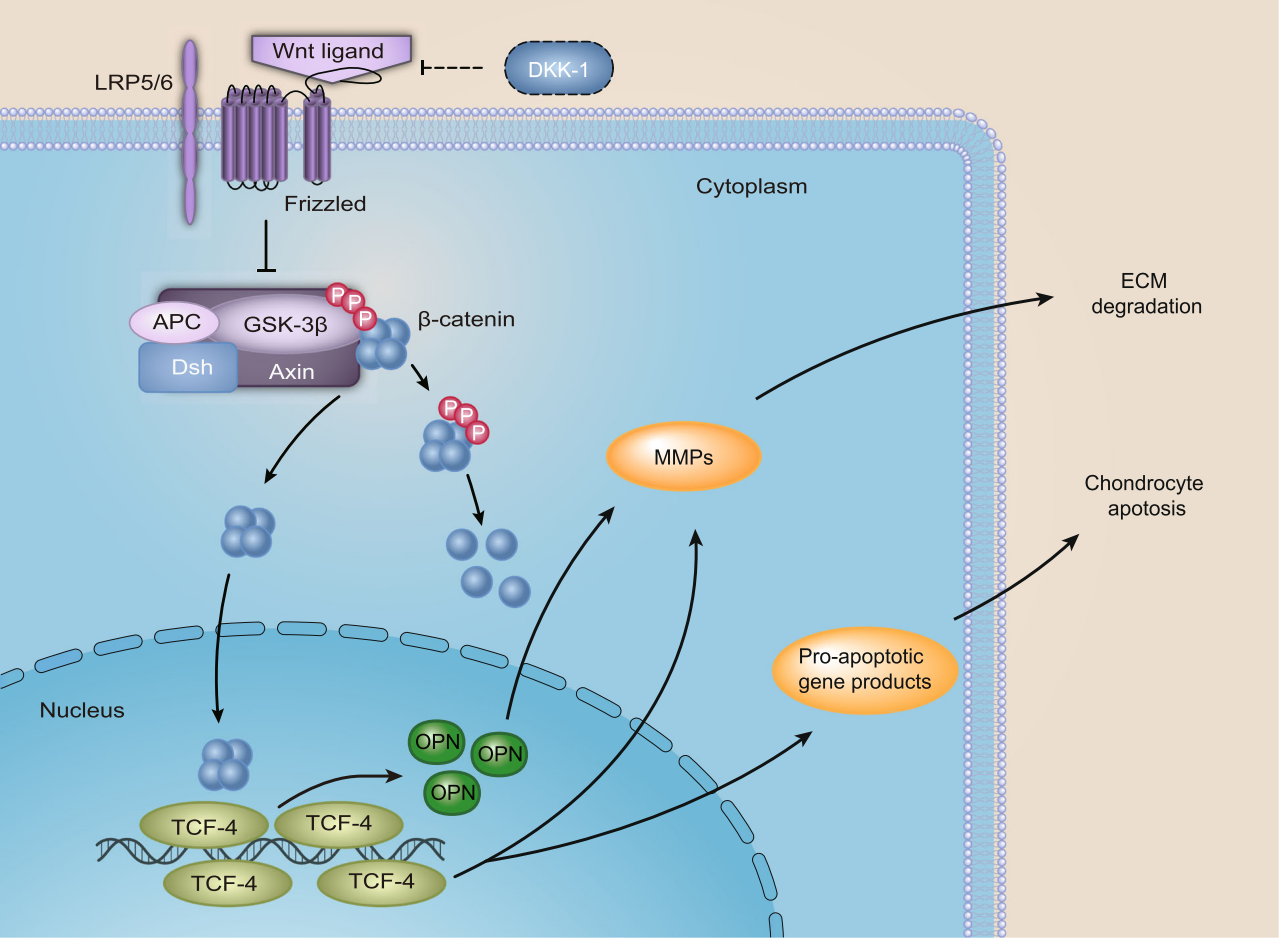
The Wnt pathway regulates OPN expression and promote OA progression

A previous study confirmed that Wnt activation in OA affects the whole joint; that Dkk-1-mediated inhibition of Wnt signaling in bone ameliorates OA in mice; that control of Dkk-1 expression ameliorates chondrocyte apoptosis, cartilage destruction, and subchondral bone deterioration in osteoarthritic knees; and that the protective effect of Dkk-1 appears to be associated with its capacity to inhibit Wnt-mediated expression of catabolic factors [21, 32, 33]. Because Dkk-1 is an inhibitor of the Wnt/β-catenin signaling pathway and is also implicated in OA, in our study, we treated chondrocytes with different doses of rDKK-1. After treatment with a beneficial concentration of rDKK-1, we used DAPI staining to assess β-catenin nuclear accumulation in chondrocytes. Our results indicated that rDKK-1 could inhibit β-catenin nuclear accumulation and that the expression of both β-catenin and OPN was decreased after chondrocytes were treated with rDKK-1. Figure 7 shows the Wnt pathway regulates OPN expression and promotes OA progression. The results were consistent with those of a previous study that showed that the addition of DKK1 markedly decreased BBR-induced β-catenin and OPN expression [34].

Taken together, our results indicate that the increase in OPN expression might be regulated by the β-catenin/TCF-4 pathway and that the Wnt/β-catenin inhibitor DKK1 could inhibit the expression of β-catenin and OPN in OA chondrocytes.

## Data Availability

The datasets used and/or analyzed during the current study are available from the corresponding author on reasonable request.

## Abbreviations

OA: Osteoarthritis
OPN: Osteopontin
Dkk-1: Dickkopf-1
rDKK-1: Recombinant Dickkopf-1
MMP13: Matrix metalloproteinase 13
PBS: Phosphate-buffered saline
DMEM/F12: Dulbecco’s modified Eagle’s medium/F12
FBS: Fetal bovine serum
Real-time Q-PCR: Real-time quantitative PCR
MTT: Colorimetric 3-(4, 5-dimethylthiazol-2-yl)-2, 5-diphenyltetrazolium bromide
DMSO: Dimethylsulfoxide
ELISA: Enzyme-linked immunosorbent assay
